# Role of the central nervous system and adipose tissue BDNF/TrkB axes in metabolic regulation

**DOI:** 10.1038/npjamd.2015.9

**Published:** 2015-10-29

**Authors:** Atsushi Nakagomi, Sho Okada, Masataka Yokoyama, Yohko Yoshida, Ippei Shimizu, Takashi Miki, Yoshio Kobayashi, Tohru Minamino

**Affiliations:** 1 Department of Cardiovascular Medicine, Chiba University Graduate School of Medicine, Chiba, Japan; 2 Department of Cardiovascular Biology and Medicine, Niigata University Graduate School of Medical and Dental Sciences, Niigata, Japan; 3 Department of Medical Physiology, Chiba University Graduate School of Medicine, Chiba, Japan; 4 PRESTO, Japan Science and Technology Agency, Saitama, Japan

## Abstract

**Background/Objectives:**

Brain-derived neurotrophic factor (BDNF) and its receptor (tropomyosin-related kinase B: TrkB, also known as Ntrk2) have a key role in central regulation of the energy balance. BDNF and TrkB are also expressed in the peripheral tissues, including adipose tissue, but their peripheral role has been unclear. Here we report on the functional significance of the adipose tissue BDNF/TrkB axis in metabolic homeostasis.

**Materials and Methods:**

To examine the role of the BDNF/TrkB axis in the central nervous system and in adipose tissue, we generated adipocyte-specific or neuron-specific BDNF/TrkB conditional knockout (CKO) mice. Then we compared the feeding behavior and metabolic profile between each type of CKO mouse and their littermates.

**Results:**

*Bdnf* expression was significantly increased in the adipose tissue of mice receiving a high-calorie diet, whereas *Ntrk2* expression was decreased. The *Bdnf*/*Ntrk2* expression ratio of adipose tissue was higher in female mice than male mice. *Fabp4*-Cre mice are widely used to establish adipocyte-specific CKO mice. However, we found that *Fabp4*-Cre-induced deletion of *Bdnf* or *Ntrk2* led to hyperphagia, obesity, and aggressiveness, presumably due to ectopic *Fabp4*-Cre mediated gene recombination in the brain. Next, we attempted to more specifically delete *Bdnf* or *Ntrk2* in adipocytes using *Adipoq*-Cre mice. Expression of *Ntrk2,* but not *Bdnf,* in the adipose tissue was reduced by *Adipoq*-Cre mediated gene recombination, indicating that adipocytes only expressed TrkB. No phenotypic changes were detected when *Adipoq*-Cre TrkB CKO mice were fed a normal diet, whereas female CKO mice receiving a high-calorie diet showed a decrease in food intake and resistance to obesity.

**Conclusions:**

The adipose tissue BDNF/TrkB axis has a substantial influence on the feeding behavior and obesity in female mice.

## Introduction

Brain-derived neurotrophic factor (BDNF) is a member of the neurotrophic factor family that shows high-affinity binding to its receptor (tropomyosin-related kinase B: TrkB, also known as Ntrk2) and has a key role in regulating neuronal survival, neuronal differentiation, and synaptic plasticity.^1–4^ BDNF and the TrkB receptor are expressed in various brain regions, including energy homeostasis centers within the hypothalamus and the hindbrain in adult animals.^5–9^ Consistent with this pattern of expression, BDNF has an essential role in regulating the body weight and energy homeostasis. Chronic infusion of BDNF into the cerebral ventricles has been shown to reduce food intake and body weight,^10,11^ whereas heterozygous *Bdnf-*deficient mice display increased locomotor activity, aggressiveness, hyperphagia, and obesity.^12,13^ In addition, mice with approximately 75% reduction of TrkB expression exhibit hyperphagia and obesity,^5^ while mice lacking neuronal *Bdnf* develop obesity and anxiety.^14^ It is thought that the anorexic actions of BDNF and TrkB are mediated by the leptin-proopiomelanocortin (POMC) signaling pathway.^5^ In humans, genome-wide association studies^15–18^ and rare reports on genetic variants of *BDNF*^19^ and *NTRK2*^20^ have shown an association between the BDNF/TrkB axis and obesity in both adults and children.

A number of studies have demonstrated that BDNF and TrkB are also expressed in non-neural tissues and have an influence on the eating behavior and metabolic homeostasis. The circulating level of BDNF is associated with obesity and diabetes mellitus in humans,^21^ while systemic administration of BDNF improves glycemic control in db/db mice.^22^ The liver and pancreatic α-cells might be involved in the underlying mechanism.^23,24^ In addition, mice with hepatocyte-specific *Bdnf* deficiency show normal food intake and body weight, but are protected against dietary metabolic abnormalities through enhanced expression of peroxisome proliferator-activated receptor α and fibroblast growth factor 21 (ref. 25). It was reported that BDNF expression is induced in the skeletal muscles by exercise and enhances muscle fat oxidation via activation of AMP-activated protein kinase.^26^ It has also been demonstrated that BDNF-expressing hematopoietic cells regulate feeding behavior and energy balance by migrating from the bone marrow to the paraventricular nucleus of the hypothalamus.^27^ Furthermore, it was reported that BDNF is expressed in adipose tissue.^28,29^ Moreover, humoral factors released by adipose tissue, collectively termed adipokines, and certain metabolites like fatty acids are involved in the regulation of energy expenditure and glucose/lipid metabolism through various systemic actions.^30–32^ Taken together, these reports suggest the possibility that the BDNF/TrkB axis in adipose tissue may have a role in the regulation of systemic metabolism. To test this hypothesis, we investigated expression of BDNF and TrkB by adipose tissue in a mouse model of dietary obesity and generated various conditional knockout (CKO) mouse models by using the Cre-loxP system. Our findings demonstrated that the adipose tissue BDNF/TrkB axis has a critical role in regulating metabolic homeostasis.

## Materials and methods

### Animal models

All animal care and experimental procedures were approved by the Chiba University review board. Mice were housed in individual cages in a temperature-controlled facility with a 12-h day/night cycle and free access to water. The mice were fed a normal chow diet (CLEA Rodent diet CE-2; CLEA Japan, Tokyo, Japan) or a high-fat/high-sucrose diet (F2HFHSD, Oriental Yeast, Suita, Japan) from 6 weeks of age. C57BL/6 mice were purchased from Japan SLC (Shizuoka, Japan). Floxed *Bdnf* mice, *Syn1*-Cre mice, and *Fabp4*-Cre mice (with a C57BL/6 background) were purchased from the Jackson Laboratory (Bar Harbor, ME, USA). Generation and genotyping of floxed *Ntrk2* mice (a gift from Luis F Parada, University of Texas Southwestern Medical Center, Dallas, TX, USA) and *Adipoq*-Cre mice (a gift from Evan D Rosen, Beth Israel Deaconess Medical Center, Boston, MA, USA) have been described previously.^33,34^

The mice were fasted for 12–14 h before being killed. For fractionation of adipose tissue, freshly isolated epididymal fat pads were dissected, minced in phosphate-buffered saline with 1% bovine serum albumin, and then incubated with 0.05 mg/ml Liberase TM (Roche, Indianapolis, IN, USA) and 50 units/ml DNase I (Sigma-Aldrich, St Louis, MO, USA) for 45 min at 37 °C in an orbital shaker with intermittent pipetting. The digested samples were filtered through a sterile 250-μm nylon mesh, after which the suspension was centrifuged at 1,000 g for 5 min to separate the adipocyte fraction from the stromal vascular fraction.

The genotypes of the mice and the effects of Cre-mediated recombination were assessed by PCR using genomic DNA harvested from the tail tips and various other tissues of mutant mice. To examine tissue-specific deletion of BDNF or TrkB in CKO mice, we analyzed male mice homozygous for the floxed allele with one copy of the Cre recombinase transgene (CKO), littermate mice homozygous for the floxed allele without the Cre recombinase transgene (littermate controls), and wild-type C57BL/6 mice using the following primers: Cre recombinase, 5′-GTTCGCAAGAACCTGATGGACA-3′ and 5′-CTAGAGCCTGTTTTGCACGTTC-3′; wild-type or floxed *Bdnf* allele, 5'-TGTGATTGTGTTTCTGGTGAC-3′, 5′-GATACATCATGGGCAGTGGA-3′; wild-type or floxed *Ntrk2* allele, 5′-ATGTACTCGTTCTACAAATCCTGC-3′, 5′-TCCAGACACATACACGTGCGTGC-3′, and 5′-CAAGAAGTCAGAGACCAGAGAGA-3′.

### RNA analysis

Total RNA was extracted from adipose tissue by using an RNeasy Plus Universal kit (Qiagen, Valencia, CA, USA) and was extracted from other tissues with RNA-Bee (Molecular Research Center, Cincinnati, OH, USA). Then reverse transcription was performed by using a QuantiTect reverse transcription kit (Qiagen), and real-time PCR was done with a LightCycler (Roche), the Taqman Universal Probe Library, and Light Cycler Master (Roche) according to the manufacturer’s instructions. For normalization of gene expression, *36B4* mRNA was used as the internal control. Data were analyzed by the 2−1ΔΔCT method. The following primers were used: *36B4*, forward 5′-GATGCCCAGGGAAGACAG-3′, reverse 5′-ACAATGAAGCATTTTGGATAATCA-3′; *Bdnf,* forward 5′-GCCTTTGGAGCCTCCTCTAC-3′, reverse 5′-GCGGCATCCAGGTAATTTT-3′; *Ntrk2*, forward 5′-CGAACCTGCAGATACCCAAT-3′, reverse 5′-TGCAGGAAAGGGTCACAGA-3′; *Ntrk2-tk*, forward 5′-TTCTGCCTGCTGGTGATGT-3′, reverse 5′-TCCAGTGGGATCTTATGAAACA-3′, *Lep*, forward 5′-CAGGATCAATGACATTTCACACA-3′, reverse 5′-GCTGGTGAGGACCTGTTGAT-3′; *Adipoq*, forward 5′-CAGGCATCCCAGGACATT-3′, reverse 5′-ACCCTTAGGACCAAGAAGACCT-3′; *Emr*, forward 5′-GGAGGACTTCTCCAAGCCTATT-3′, reverse 5′-AGGCCTCTCAGACTTCTGCTT-3′; *Tnf*, forward 5′-TGCCTATGTCTCAGCCTCTTC-3′, reverse 5′-GAGGCCATTTGGGAACTTCT-3′.

### Measurement of body weight and food intake

Body weight was measured every 2 weeks. Food intake was assessed by housing mice individually. The animals were fed a normal diet or a high-calorie diet *ad libitum*, and the amount of residual food was weighed for a total of 7 days. In pair-feeding experiments, the same amount of high-calorie diet was given to CKO mice and their littermates each day.

### Assessment of locomotor activity

Locomotor activity was assessed by using an implantable intra-abdominal radiofrequency probe and receiver (TA10TA-F20 and RPC-1, Data Sciences International, St Paul, MN, USA). Mice (14–16 weeks old) were placed individually into cages and baseline locomotor activity was monitored. Data were sampled in the continuous mode and analyzed by Dataquest ART2.1.

### CT scanning

Adipose tissue was examined by CT (LaTheta, Aloka, Tokyo, Japan) according to the manufacturer’s protocol. We obtained CT scans at 2 mm intervals from diaphragm to the floor of the pelvic cavity.

### Laboratory tests

To perform the glucose tolerance test, mice were fasted for 16 h and D-glucose (2 g/kg body weight) was administered by intraperitoneal injection at 1500 h. For the insulin tolerance test, mice were given an intraperitoneal injection of human insulin (1 U/kg body weight) at 1500 h without fasting. In both the tests, blood was collected from the tail vein at 0, 15, 30, 60, and 120 min after administration of glucose or insulin. Then blood glucose concentrations were measured with a Glutest Mint (Sanwa Kagaku Kenkyusho, Nagoya, Japan). Insulin, leptin, and adiponectin were measured by enzyme-linked immunosorbent assay-based immunoassay kits (insulin: Morinaga Institute of Biological Science, Yokohama, Japan; leptin: R&D Systems, Minneapolis, MN, USA; adiponectin: Otsuka Pharmaceutical, Tokyo, Japan) according to the manufacturer’s instruction. Free fatty acid levels were measured by Oriental Yeast.

### Statistical analysis

Results are shown as the mean±s.e.m. Differences between groups were examined by Student’s *t*-test for the comparison of mean values. In all the analyses, *P*<0.05 was considered statistically significant.

## Results

### Tissue expression of *Bdnf* and *Ntrk2*

**Figure 1:**
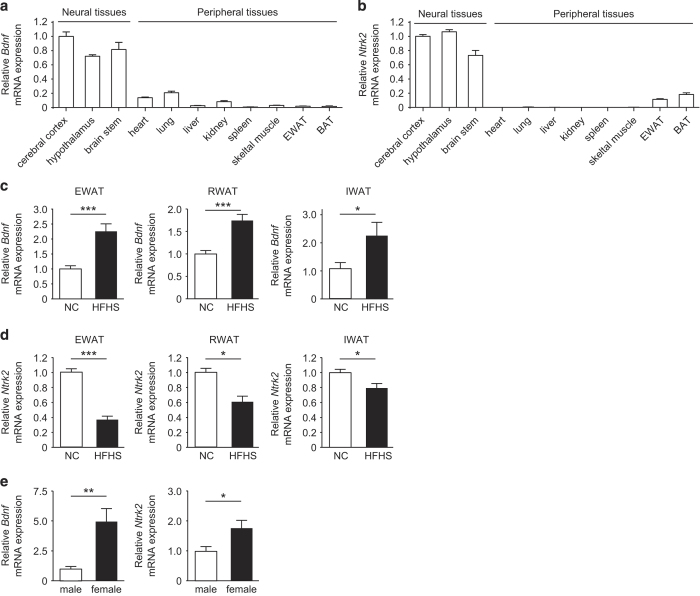
Expression of *Bdnf* and *Ntrk2* in peripheral tissues. (**a** and **b**) Relative expression of *Bdnf* (**a**) and *Ntrk2* (**b**) of mice aged 16 weeks was determined by real-time PCR. Expression in various tissues is shown relative to that in the cerebral cortex; *n*=5. EWAT, epididymal white adipose tissue; BAT, brown adipose tissue. (**c** and **d**) Expression of *Bdnf* (**c**) and *Ntrk2* (**d**) in epididymal white adipose tissue (EWAT), perinephric white adipose tissue (RWAT), and inguinal white adipose tissue (IWAT) assessed by real-time PCR in 16-week-old mice fed normal chow (NC) or a high-fat/high-sucrose (HFHS) diet; *n*=9–10 for EWAT, *n*=4 for RWAT, and *n*=4 for IWAT. (**e**) Real-time PCR analysis of *Bdnf* and *Ntrk2* expression in the epididymal adipose tissue of 21–25-week-old male and female mice fed normal chow; *n*=9. *P<0.05, **P<0.01, ***P<0.001. Data are shown as the mean±s.e.m.

We first examined the expression of *Bdnf* and *Ntrk2* in various tissues of mice fed a normal diet. *Bdnf* was highly expressed in the brain, whereas intermediate expression was found in the heart, lung, and kidney (Figure 1a). *Bdnf* was also expressed in adipose tissue, although its expression was much lower than in neural tissues. In addition, *Ntrk2* (the gene coding for TrkB) was expressed by adipose tissue at the highest level among the peripheral tissues that we examined (Figure 1b). To investigate the role of the adipose tissue BDNF/TrkB axis in obesity, we examined *Bdnf* and *Ntrk2* expression in mice with dietary obesity owing to feeding with a high-fat/high-sucrose (HFHS) diet. *Bdnf* expression was markedly upregulated in the adipose tissue of these obese mice (Figure 1c). Unlike *Bdnf*, expression of *Ntrk2* was significantly downregulated in the adipose tissue of obese mice (Figure 1d). No significant changes of *Bdnf* or *Ntkr2* expression were observed in the other peripheral tissues (Supplementary Figure S1A and 1B). We next analyzed gender differences of *Bdnf* and *Ntrk2* expression in adipose tissue, revealing that female mice showed higher expression of both *Bdnf* and *Ntrk2* than male mice (Figure 1e). On the basis of these findings and previous observations,^28,29^ we speculated that the adipose tissue BDNF/TrkB axis might have a role in regulating metabolic homeostasis, and that its role was influenced by gender.

### Mice with adipocyte-specific *Bdnf* or *Ntrk2* deficiency exhibit obesity and hyperphagia

**Figure 2:**
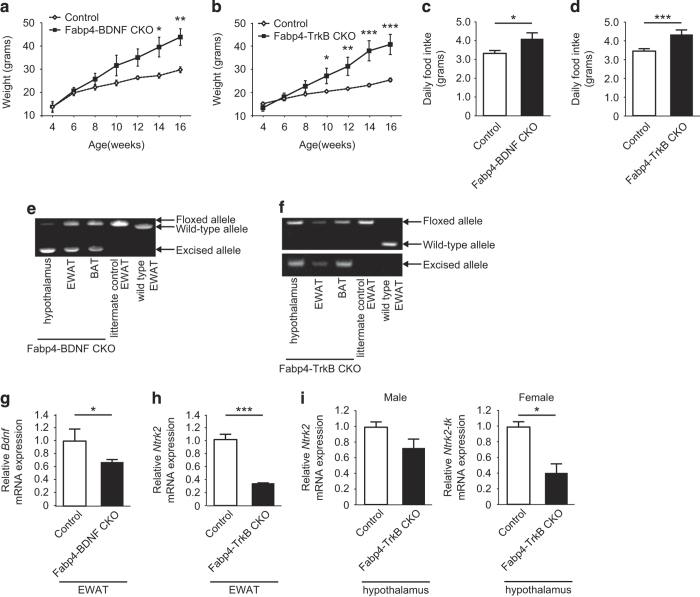
Obesity and hyperphagia in mice with adipocyte-specific deficiency of *Bdnf* or *Ntrk2* developed using *Fabp4*-Cre mice. (**a** and **b**) Body weight of Fabp4-BDNF CKO mice (**a**) and Fabp4-TrkB CKO mice (**b**) compared with their littermate controls (Control); *n*=3 for **a**, *n*=7–11 for **b**. *P<0.05, **P<0.01, ***P<0.001. (**c** and **d**) Food intake of Fabp4-BDNF CKO mice (**c**) and Fabp4-TrkB CKO mice (**d**) compared with their littermate controls (Control) at 8–12 weeks of age; *n*=4–6 for **c**, *n*=6–8 for **d**. *P<0.05, ***P<0.001. (**e** and **f**) PCR analysis of genomic DNA isolated from the hypothalamus, epididymal white adipose tissue (EWAT), and brown adipose tissue (BAT) of Fabp4-BDNF CKO mice (**e**) and Fabp4-TrkB CKO mice (**f**), and from EWAT of their littermate controls and wild-type mice. Littermate controls were homozygous for the floxed *Bdnf* (**e**) or *Ntrk2* (**f**) allele, but did not carry Cre recombinase. (**g**) Real-time PCR analysis of *Bdnf* expression by EWAT in Fabp4-BDNF CKO mice and their littermate controls (Control); *n*=3. *P<0.05. (**h** and **i**) Real-time PCR analysis of *Ntrk2* expression in EWAT (**h**) and the hypothalamus (**i**) of Fabp4-TrkB CKO mice and their littermate controls (Control); *n*=3–4. *P<0.05, ***P<0.001. Data are shown as the mean±s.e.m. CKO, conditional knockout.

To study the role of the BDNF/TrkB axis in adipose tissue, we generated mice lacking *Bdnf* or *Ntrk2* in adipocytes by breeding *Bdnf*^flox/flox^ or *Ntrk2*^flox/flox^ mice with *Fabp4*-Cre mice (Fabp4-BDNF CKO mice and Fabp4-TrkB CKO mice, respectively). Both lines of mice exhibited age-dependent obesity (Figure 2a and b), hyperphagia (Figure 2c and d), and aggressiveness (data not shown). These phenotypic changes suggested that the CKO mice had abnormalities of the central nervous system. Although Fabp4 was originally identified as an adipocyte-specific protein, recent studies have shown that it is also expressed by other types of cells, including neurons.^35^ As BDNF acts on the hypothalamus to regulate energy homeostasis,^5,9,11^ we investigated the specificity of *Fabp4*-Cre-mediated gene recombination. Genomic PCR detected the excised allele of *Bdnf*^flox/flox^ mice in the hypothalamus as well as in adipose tissue of Fabp4-BDNF CKO mice (Figure 2e). Likewise, the excised allele of *Ntrk2*^flox/flox^ mice was observed in the hypothalamus of Fabp4-TrkB CKO mice (Figure 2f). Adipose tissue expression of both *Bdnf* and *Ntrk2* was markedly reduced in these CKO mice (Figure 2g and h). Consistent with the genomic PCR data, hypothalamic expression of *Ntrk2* was downregulated in Fabp4-TrkB CKO mice (Figure 2i), indicating that *Fabp4*-Cre-mediated gene recombination in the hypothalamus could affect the metabolic phenotype of Fabp4-TrkB CKO mice.

### Neuronal *Bdnf* deficiency is associated with a similar phenotype to that of Fabp4-BDNF/TrkB CKO mice

To examine whether *Fabp4*-Cre-mediated recombination in the brain accounted for the obese phenotype of Fabp4-BDNF/TrkB CKO mice, we next generated mice lacking neuronal *Bdnf* by breeding *Bdnf*^*flox/flox*^ mice with *Syn1-*Cre mice (Syn1-BDNF CKO mice). We confirmed by genomic PCR that *Syn1*-Cre-mediated recombination was only detected in the nervous system (Supplementary Figure S2A), in agreement with a previous report.^36^ *Bdnf* expression was only decreased in the brains of Syn1-BDNF CKO mice (Supplementary Figure S2B). Syn1-BDNF CKO mice exhibited a higher body weight compared with their littermate controls (Supplementary Figure S2C), which was associated with hyperphagia (Supplementary Figure S2D) and increased locomotor activity (Supplementary Figure S2E). These results suggested that *Fabp4*-Cre-mediated gene recombination in the brain had an influence on the metabolism of Fabp4- BDNF/TrkB CKO mice.

### Adipocyte-specific *Bdnf/Ntrk2* deletion does not cause obesity in *Adipoq*-Cre mice

**Figure 3:**
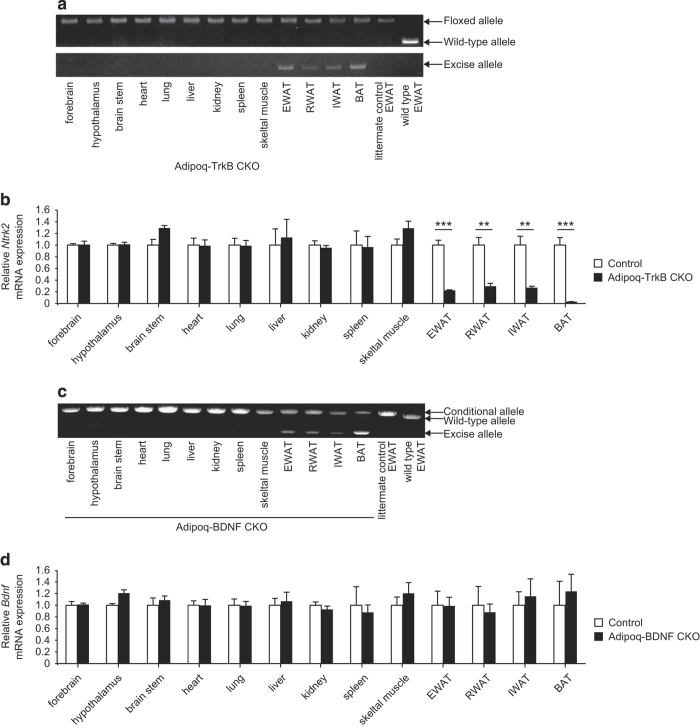
Genomic recombination and gene expression in adipocyte-specific Bdnf/Ntrk2 deletion models created using *Adipoq*-Cre mice. (**a**) PCR analysis of genomic DNA isolated from various tissues of Adipoq-TrkB CKO mice and from epididymal white adipose tissue (EWAT) of their littermate controls and wild-type mice. Littermate controls were homozygous for the floxed *Ntrk2* allele, but did not carry Cre recombinase. (**b**) Real-time PCR analysis of *Ntrk2* expression in various tissues of Adipoq-TrkB CKO mice and their littermate controls (Control); *n*=5. **P<0.01, ***P<0.001. (**c**) PCR analysis of genomic DNA isolated from various tissues of Adipoq-BDNF CKO mice and from epididymal white adipose tissue (EWAT) of their littermate controls and wild-type mice. Littermate controls were homozygous for the floxed *Bdnf* allele, but did not carry Cre recombinase. (**d**) Real-time PCR analysis of *Bdnf* expression in various tissues of Adipoq-BDNF CKO mice and their littermate controls (Control); *n*=5. Data are shown as the mean±s.e.m. CKO, conditional knockout.

To investigate the possibility of genetic recombination occurring in the brain, we generated a second line of adipocyte-specific Bdnf/Ntrk2 CKO mice by breeding *Bdnf*^flox/flox^ or *Ntrk2*^flox/flox^ mice with *Adipoq*-Cre mice (Adipoq-BDNF CKO mice and Adipoq-TrkB CKO mice, respectively). It has been reported that Cre expression driven by the *Adipoq* promoter leads to recombination that exclusively affects adipocytes,^34,37^ and we confirmed that Adipoq-TrkB CKO mice exhibited adipose tissue-specific recombination (Figure 3a) and that *Ntrk2* was specifically deleted from adipose tissue (Figure 3b). Although Adipoq-BDNF CKO mice displayed adipose tissue-specific recombination (Figure 3c), *Bdnf* expression was unexpectedly not suppressed in their adipose tissue (Figure 3d), suggesting that adipocytes do not contribute to the production of BNDF by adipose tissue. In contrast, the pattern of expression in Adipoq-TrkB CKO mice supported the concept that *Ntrk2* is predominantly expressed by adipocytes in the adipose tissue. Consistent with these results, real-time PCR analysis of isolated adipocytes and the stromal vascular fraction from adipose tissue revealed that *Bdnf* expression was elevated in the stromal vascular fraction, while there was higher expression of *Ntrk2* in the adipocyte-rich fraction (Supplementary Figure S3).

**Figure 4:**
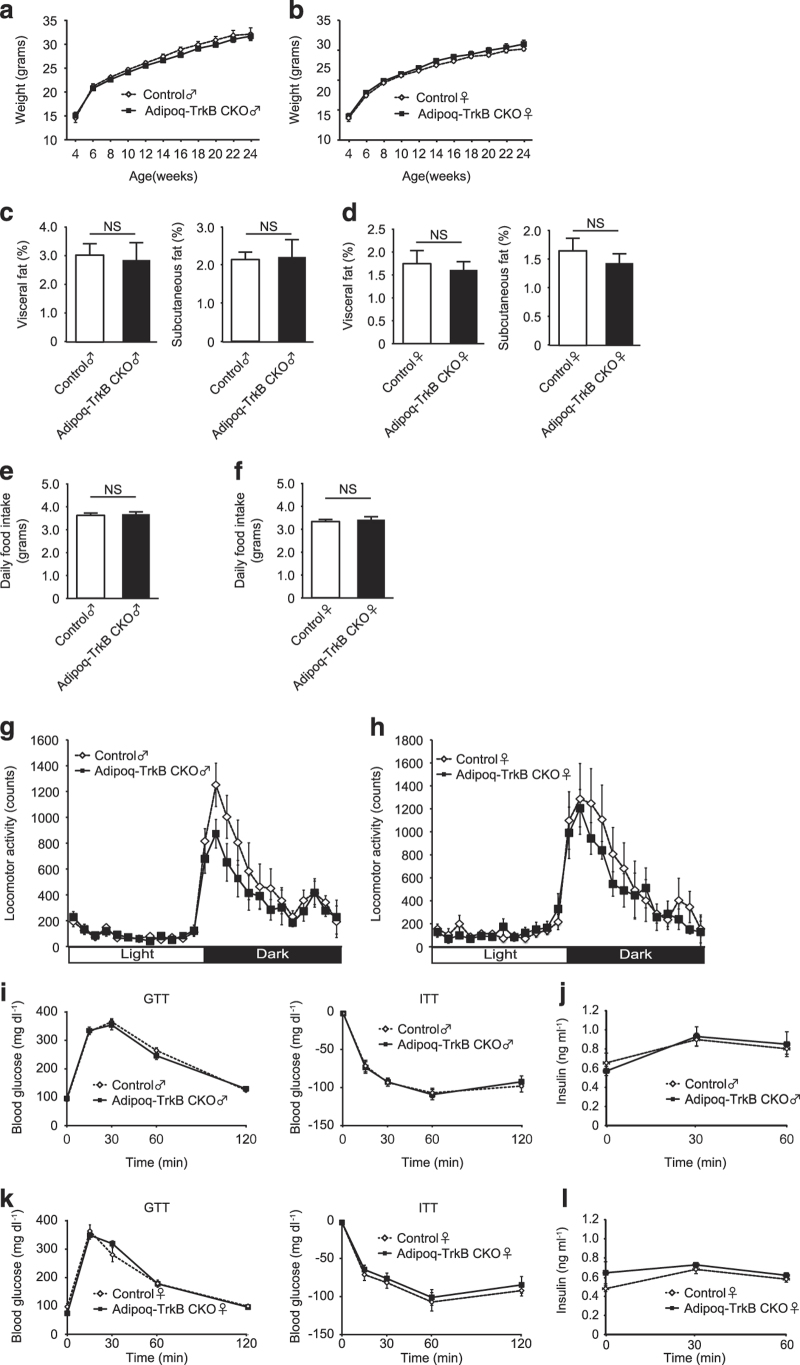
Adipocyte-specific deletion of Ntrk2 does not cause obesity. (**a** and **b**) Body weight of Adipoq-TrkB CKO male mice (**a**) and female mice (**b**) receiving a normal diet compared with their littermate controls (Control); *n*=11–12 for **a**, *n*=10–11 for **b**. (**c** and **d**) CT analysis of Adipoq-TrkB CKO male mice (**c**) and female mice (**d**) receiving a normal diet compared with their littermate controls (Control) at 12–14 weeks of age. The percent fat tissue/body weight ratio is shown for visceral fat and subcutaneous fat; *n*=6 for **c**, *n*=9–10 for **d**. (**e** and **f**) Food intake of Adipoq-TrkB CKO male mice (**e**) and female mice (**f**) receiving a normal diet compared with their littermate controls (Control) at 8–12 weeks of age; *n*=9–10 for **e**, *n*=9–10 for **f**. (**g** and **h**) Locomotor activity of Adipoq-TrkB CKO male mice (**g**) and female mice (**h**) receiving a normal diet compared with their littermate controls (Control) at 14–16 weeks of age; *n*=6 for **g**, *n*=6 for **h**. (**i**) Glucose tolerance test (GTT) and insulin tolerance test (ITT) in Adipoq-TrkB CKO male mice receiving a normal diet compared with their littermate controls (Control) at 12–14 weeks of age; *n*=9–13 for GTT, *n*=10–13 for ITT. (**j**) Plasma insulin level during the GTT shown in Figure 5i; *n*=9–10. (**k**) GTT and ITT in Adipoq-TrkB CKO female mice receiving a normal diet compared with their littermate controls (Control) at 12–14 weeks of age; *n*=15–16 for GTT, *n*=7–10 for ITT. (**l**) Plasma insulin level during the GTT shown in Figure 5k; *n*=7–10. Data are shown as the mean±s.e.m. CKO, conditional knockout.

We then focused on TrkB in adipose tissue to investigate the role of the BDNF/TrkB axis in metabolism. As decreased expression of TrkB by adipose tissue was associated with obesity (Figure 1d), we hypothesized that BDNF might positively regulate energy metabolism by acting on adipocyte TrkB. We monitored physiological parameters in male and female mice on a normal diet, revealing that Adipoq-TrkB CKO mice and their littermate controls showed no differences in body weight (Figure 4a and b), fat mass (Figure 4c and d), food intake (Figure 4e and f), or locomotor activity (Figure 4g and h). Consistent with the results for *Bdnf* expression, Adipoq-Bdnf CKO mice did not display any changes in the body weight, fat mass, food intake, and locomotor activity (data not shown). To assess the role of the adipose tissue BDNF/TrkB axis in glucose homeostasis, we performed glucose tolerance and insulin tolerance tests, and we measured plasma insulin levels during the glucose tolerance test. Normal glucose metabolism was observed in mice of both sexes (Figure 4i–l). Taken together, these results suggest that adipose tissue *Ntrk2* only has a minor role in regulating eating behavior and metabolism in mice receiving a normal diet.

### Adipoq-TrkB CKO female mice on a high-calorie diet show decreased food intake and resistance to obesity

**Figure 5:**
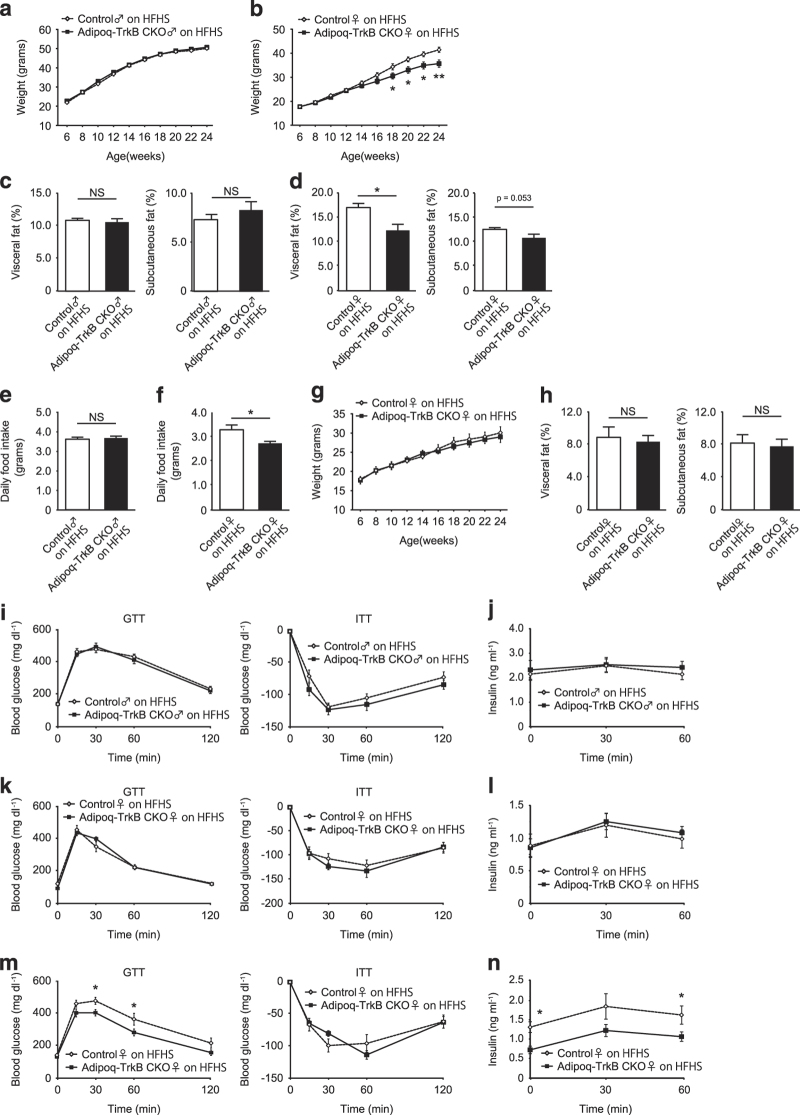
Adipoq-TrkB CKO female mice on a high-calorie diet show decreased food intake and resistance to obesity. (**a** and **b**) Body weight of Adipoq-TrkB CKO male mice (**a**) and female mice (**b**) receiving a high-fat/high-sucrose (HFHS) diet compared with their littermate controls (Control); *n*=14−16 for **a**, *n*=26–33 for **b**. *P<0.05, **P<0.01. (**c** and **d**) CT analysis of Adipoq-TrkB CKO male mice (**c**) and female mice (**d**) receiving the HFHS diet compared with their littermate controls (Control) at 24 weeks of age. The percent fat tissue/body weight ratio is shown for visceral fat and subcutaneous fat; *n*=5 for **c**, *n*=7 for **d**. *P<0.05. (**e** and **f**) Food intake of Adipoq-TrkB CKO male mice (**f**) and female mice (**g**) receiving the HFHS diet compared with their littermate controls (Control) at 14 weeks of age; *n*=10–13 for **f**, *n*=14–16 for **g**. *P<0.05. (**g**) Pair-feeding experiments in Adipoq-TrkB CKO female mice receiving the HFHS diet compared with their littermate controls (Control); *n*=6. (**h**) CT analysis of Adipoq-TrkB CKO female mice receiving the HFHS diet compared with their littermate controls (Control) at 12–14 weeks of age. The percent fat tissue/body weight ratio is shown for visceral fat and subcutaneous fat; *n*=7. (**i**) Glucose tolerance test (GTT) and insulin tolerance test (ITT) in Adipoq-TrkB CKO male mice receiving the HFHS diet compared with their littermate controls (Control) at 12–14 weeks of age; *n*=17–18 for GTT, *n*=15–18 for ITT. (**j**) Plasma insulin level during the GTT shown in Figure 6i; *n*=12–15. (**k**) Glucose tolerance test (GTT) and insulin tolerance test (ITT) in Adipoq-TrkB CKO male mice receiving the HFHS diet compared with their littermate controls (Control) at 12–14 weeks of age; *n*=10–13 for GTT, *n*=10 for ITT. (**l**) Plasma insulin level during the GTT shown in Figure 6k; *n*=14–15. (**m**) Glucose tolerance test (GTT) and insulin tolerance test (ITT) in Adipoq-TrkB CKO male mice receiving the HFHS diet compared with their littermate controls (Control) at 24–26 weeks of age; *n*=14–15 for GTT, *n*=12–13 for ITT. *P<0.05. (**n**) Plasma insulin level during the GTT shown in Figure 6m; *n*=10. *P<0.05. Data are shown as the mean±s.e.m. CKO, conditional knockout.

We next examined the effect of a high-calorie diet on the metabolic phenotype of Adipoq-TrkB CKO mice. We found that female, but not male, Adipoq-TrkB CKO mice fed a high-calorie diet had a lower body weight (Figure 5a and b) and less accumulation of adipose tissue (Figure 5c and d) than their littermate controls. Adipoq-TrkB CKO female mice (but not male mice) showed a decrease in food intake (Figure 5e and f), suggesting that TrkB deficiency in adipose tissue leads to weight loss due to lower calorie intake. To test this hypothesis, we next performed a pair-feeding experiment. This showed that providing equal food intake diminished the difference in body weight between female Adipoq-TrkB CKO mice and their littermate controls (Figure 5g), suggesting that TrkB may have a role in regulating food intake. Thus, we assessed glucose metabolism in Adipoq-TrkB CKO female mice at the ages of 12–14 weeks and at 24–26 weeks. There were no differences of fat mass (Figure 5h), glucose tolerance, insulin tolerance, and the plasma insulin level between Adipoq-TrkB CKO mice and their littermate controls at 12–14 weeks (Figure 5i–l). In contrast, Adipoq-TrkB CKO female mice aged 24–26 weeks exhibited improvement of glucose tolerance and had lower insulin levels (Figure 5m and n). HOMA-IR data indicated that Adipoq-TrkB CKO mice had superior insulin sensitivity compared with control mice (14.1±1.7 vs. 6.2±1.2, *P*<0.01). Although blood glucose levels were significantly lower in Adipoq-TrkB CKO mice than in their littermate controls throughout the ITT (Supplementary Figure S4), there were no differences in the relative decrease of glucose between these two groups (Figure 5m). This suggested that the results of the ITT could have been affected by the lower body weight of Adipoq-TrkB CKO mice.

**Figure 6:**
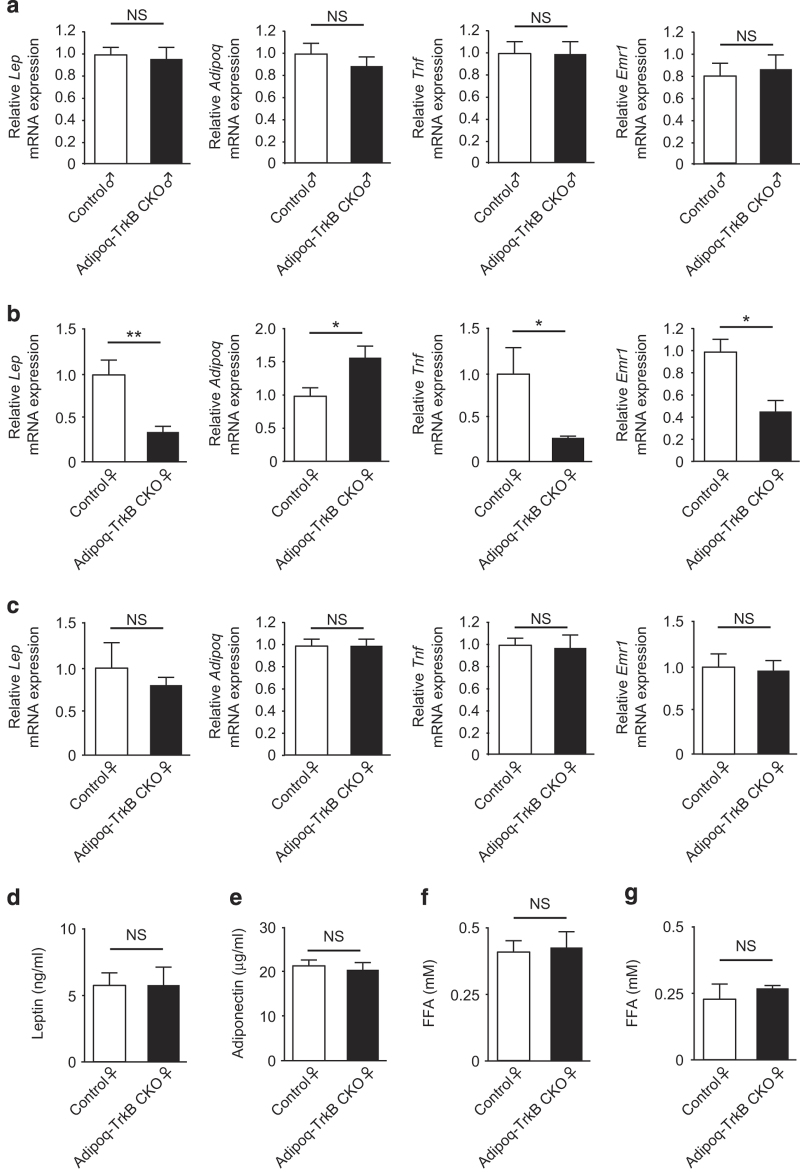
Adipokine expression in adipose tissue of Adipoq-TrkB CKO female mice. (**a** and **b**) Real-time PCR analysis of *Lep*, *Adipoq*, *Tnf*, and *Emr1* expression in epididymal adipose tissue from Adipoq-TrkB CKO male (**a**) and female (**b**) mice and their littermate controls receiving the HFHS diet at 24 weeks of age; *n*=4–5. (**c**) Real-time PCR analysis of *Lep*, *Adipoq*, *Tnf*, and *Emr1* expression in epididymal adipose tissue from Adipoq-TrkB CKO female mice and their littermate controls receiving the HFHS diet at 12 weeks of age; *n*=4–5. (**d** and **e**) Serum leptin (**d**) and adiponectin (**e**) levels in Adipoq-TrkB CKO female mice and their littermate controls receiving the HFHS diet at 12 weeks of age; *n*=7. (**f** and **g**) Serum free fatty acids (FFA) under fasted (**f**) and fed (**g**) conditions in Adipoq-TrkB CKO female mice and their littermate controls receiving the HFHS diet at 12 weeks of age; *n*=4–7. *P<0.05. Data are shown as the mean±s.e.m. CKO, conditional knockout; HFHS, high-fat/high-sucrose.

Next, we analyzed adipokine expression. At the age of 24 weeks, female Adipoq-TrkB CKO mice, but not male mice, showed lower expression of *Lep* (the gene coding for leptin), *Tnf*, and *Emr1* (a marker of macrophages) than their littermate controls (Figure 6a and b). In addition, the expression of adiponectin in adipose tissue was significantly higher in 24-week-old female Adipoq-TrkB CKO mice, but not male mice, compared with their littermate controls (Figure 6a and b). In contrast, there were no significant differences in the expression of these molecules between female Adipoq-TrkB CKO mice and their littermate controls at 12 weeks of age when their body weight did not differ (Figure 6c). These results suggested that reduction of adiposity owing to decreased food intake secondary to deletion of TrkB in adipose tissue led to attenuation of the dysregulated expression of adipokines and adipose tissue inflammation, which could account for the improvement of glucose metabolism in Adipoq-TrkB CKO female mice aged 24–26 weeks. To further address the potential mechanisms underlying the decrease in food intake, we measured circulating levels of leptin, adiponectin, and free fatty acids in Adipoq-TrkB CKO mice and their littermate controls aged 12 weeks. There were no differences between these two groups (Figure 6d–g), suggesting that the adipose tissue BDNF/TrkB axis influences feeding behavior via an unconventional pathway.

## Discussion

In the present study, we investigated the pathophysiological role of BDNF/TrkB signaling in peripheral tissues. We found significant changes of *Bdnf* and *Ntrk2* expression in the adipose tissue of mice with dietary obesity, with *Bdnf* being upregulated and *Ntrk2* being downregulated. Data from adipocyte-specific CKO models indicated that *Ntrk2* is predominantly expressed by adipocytes, while *Bdnf* is mainly expressed by other cells. Adipocyte-specific deletion of *Ntrk2* in female mice, but not male mice, receiving a high-calorie diet led to reduced food intake and less weight gain compared with littermate controls owing to a decrease in fat mass, indicating a potential role of adipose tissue BDNF/TrkB signaling in regulating both appetite and fat storage.

Deletion of TrkB mediated by *Adipoq*-Cre led to changes in the eating behavior without any alteration of neuronal *Ntrk2* expression. As it is known that food intake is regulated by the hypothalamic feeding center,^38^ our results suggested that the adipose tissue BDNF/TrkB axis remotely regulates feeding behavior in obese mice. It is noteworthy that the hypothalamic BDNF/TrkB axis negatively regulates the appetite, in contrast to positive regulation by the adipose tissue BDNF/TrkB axis. The impact of the adipose tissue BDNF/TrkB axis on feeding behavior appears to be relatively small, as deletion of the hypothalamic and adipose BDNF/TrkB axes by *Fabp4*-Cre-mediated recombination led to an increase in body weight with hyperphagia. It has been reported that adipose tissue regulates feeding behavior by secreting adipokines like leptin and adiponectin^30,39^ or by releasing metabolites such as fatty acids.^40^ However, there were no changes in the circulating levels of these molecules before the onset of obesity in female Adipoq-TrkB CKO mice. Although we observed significant changes of adipokine expression in Adipoq-TrkB CKO mice at 24 weeks of age, these changes were probably secondary to reduced adiposity owing to a decrease in food intake. Thus, metabolic regulation by the adipose tissue BDNF/TrkB axis could involve other adipokines and metabolites, but the molecular mechanisms involved remain to be determined. Future metabolomic analyses could help to identify the molecular pathways regulating communication between adipose tissue and the brain.

It is unclear why deletion of adipose tissue TrkB had different effects on the feeding behavior of male and female mice. Estrogen regulates the expression of BDNF and TrkB and has functions that overlap those of BDNF in the central nervous system.^41^ In addition, the plasma level of BDNF differs between men and women^42,43^ and is positively correlated with the estrogen level in female subjects.^44^ In the present study, we observed higher levels of adipose *Bdnf* and *Ntrk2* expression in female mice than in male mice. These data suggest that there are gender differences in the biological activity of the central and peripheral BDNF/TrkB axes. It is widely accepted that estrogen regulates food intake and adiposity via the estrogen receptor.^45^ Because adipocytes express the estrogen receptor,^46^ it may mediate the influence of estrogen on food intake and adiposity.^47,48^ Our results also raise the possibility that the BDNF/TrkB axis and estrogen may interact in adipocytes, as well as in the brain, for the regulation of appetite and fat accumulation.

It has been reported that BDNF is expressed by adipocytes^29^ and might influence energy metabolism as one of the adipokines.^49^ Although we confirmed that *Bdnf* expression was increased in the adipose tissue of obese mice, we did not detect a significant decrease of *Bdnf* expression in the adipose tissue of Adipoq-BDNF CKO mice. There is evidence that Cre expression driven by the *Fabp4* promoter leads to recombination in macrophages as well as in adipocytes,^50^ and it has been reported that macrophages express *Bdnf,*^51^ suggesting that macrophages may be the main source of BDNF in adipose tissue. In agreement with this idea, we predominantly detected *Bdnf* expression in the stromal vascular fraction of adipose tissue from obese mice. Accordingly, it is possible that an increase of infiltrating macrophages accounts for upregulation of *Bdnf* expression in the adipose tissue of obese mice. Moreover, the *Fabp4*-Cre phenotypic features of hyperphagia and obesity could be at least partly explained by deletion of *Bdnf* from macrophages, because BDNF-producing hematopoietic cells have been reported to regulate appetite and energy balance by migrating from bone marrow to the hypothalamus.^27^ Pre-adipocytes are another possible source of BDNF because *Bdnf* expression was reported to decrease during adipocyte differentiation^29^ and Adipoq-mediated recombination may be less efficient in pre-adipocytes than in adipocytes. It has been reported that the circulating level of BDNF is affected by various factors in humans,^21,42,43^ but we could not detect BDNF in mice by immunoassay, presumably owing to a species difference.^52^ Neurons innervating adipose tissue could be another source of BDNF, but the neuronal contribution appears to be small because adipose tissue *Bdnf* expression was not downregulated in Syn1-BDNF CKO mice.

In summary, we found that BDNF/TrkB expression in adipose tissue was altered by a high-calorie diet. In contrast to the known role of the BDNF/TrkB axis in the central nervous system, deletion of TrkB from adipocytes led to decreased food intake and reduced accumulation of fat in female mice fed a high-calorie diet, thereby improving various metabolic abnormalities associated with obesity. Thus, inhibition of the adipose tissue BDNF/TrkB axis may be a potential strategy for the treatment of dietary metabolic abnormalities, especially in females.
