# Supplementary material for: Role of the central nervous system and adipose tissue BDNF/TrkB axes in metabolic regulation
Source: NPJ Aging Mech Dis. 2015 Oct 29;1:15009–. doi: 10.1038/npjamd.2015.9 (PMC5514989; doi:10.1038/npjamd.2015.9)
Supplement: Supplementary Figures [file npjamd20159-s1.pdf]

Supplementary Figure S1

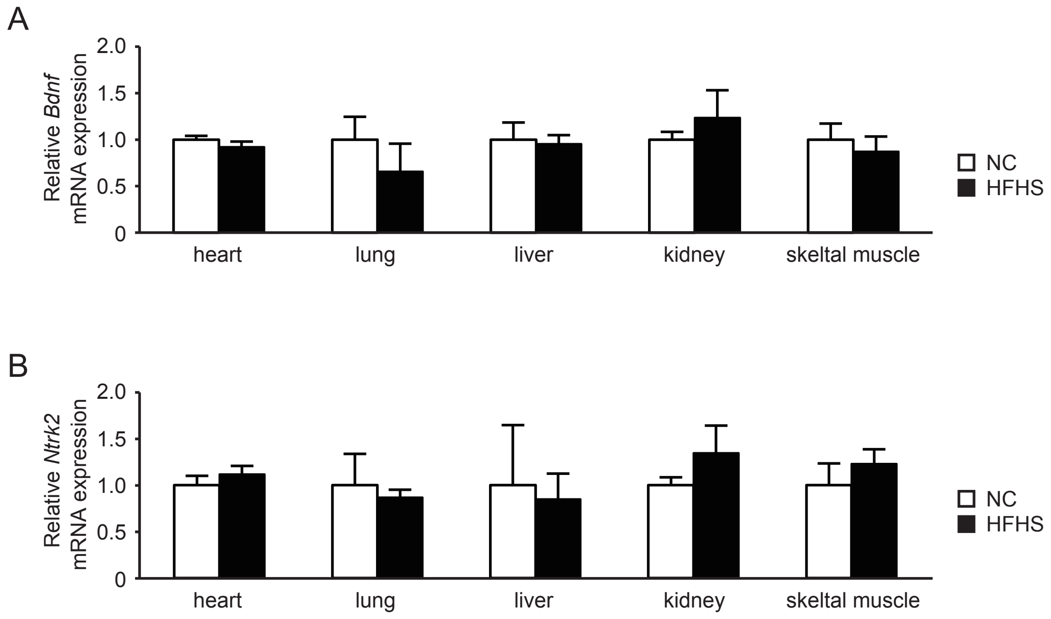

Supplementary Figure S1. Bdnf and Ntrk2 expression in the peripheral tissues of mice with dietary obesity. (A, B) Expression of Bdnf (A) and Ntrk2 (B) in several peripheral tissues assessed by real-time PCR in 16-week-old mice fed normal chow (NC) or a high-fat/high-sucrose (HFHS) diet.

Supplementary Figure S2

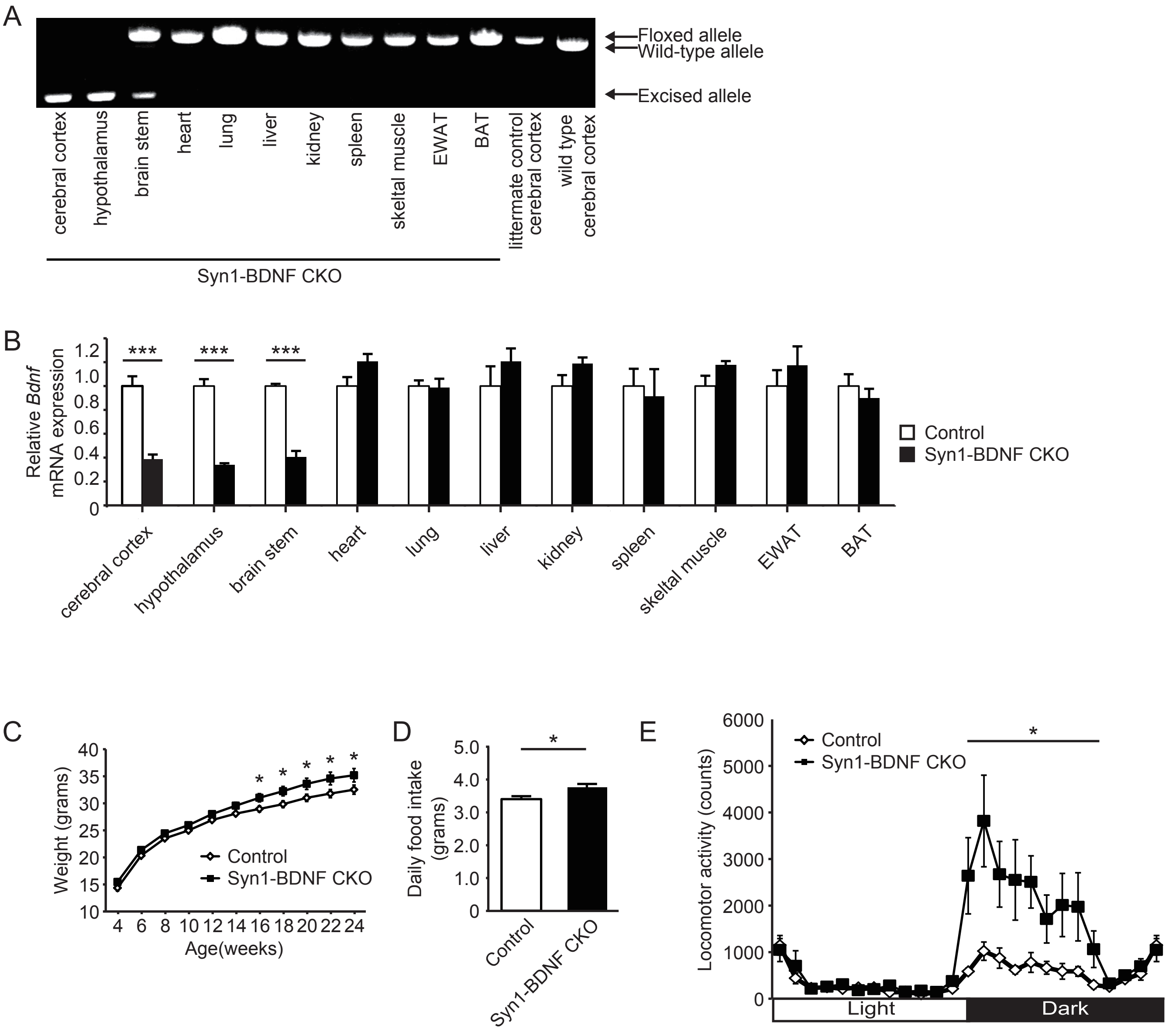

Supplementary Figure S2. Neuronal Bdnf deficiency leads to similar phenotypes to those of Fabp4-BDNF/TrkB CKO mice.
(A) PCR analysis of genomic DNA isolated from various tissues of Syn1-BDNF CKO mice and from the cerebral cortex of their littermate controls and wild-type mice. Littermates were homozygous for the floxed Bdnf allele, but did not carry Cre recombinase.
(B) Real-time PCR analysis of Bdnf expression in various tissues of Syn1-BDNF CKO mice and their littermate controls (Control). n = 5. ***$p < 0.001$.
(C) Body weight of Syn1-BDNF CKO mice and their littermate controls (Control). n = 22–25. *$p < 0.05$.
(D) Food intake of Syn1-BDNF CKO mice and their littermate controls (Control) at 8–12 weeks of age. n = 12–14. *$p < 0.05$.
(E) Locomotor activity of Syn1-BDNF CKO mice and littermate controls (Control) at 14–16 weeks of age. n = 8. *$p < 0.05$. Data are shown as the mean ± SEM.

Supplementary Figure S3

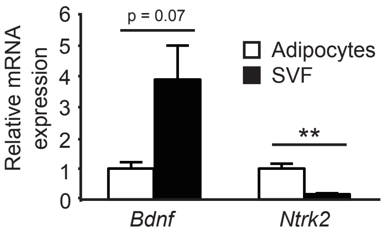

Supplementary Figure S3. Bdnf expression was elevated in the stromal vascular fraction, while expression of Ntrk2 was increased in adipocytes.Expression of Bdnf and Ntrk2 in adipocytes and the stromal vascular fraction (SVF) was assessed by real-time PCR.
n = 3-5. **p < 0.01. Data are shown as the mean ± SEM.

Supplementary Figure S4

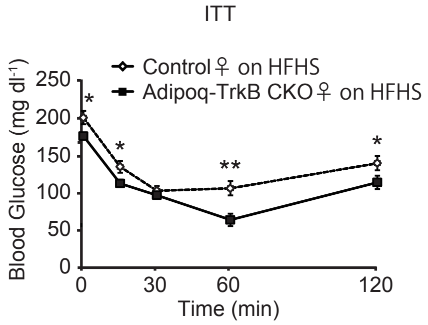

Supplementary Figure S4. Blood glucose levels in Adipoq-TrkB CKO mice and their littermate controls during ITT

n = 12-13. *p < 0.05, **p < 0.01. Data are shown as the mean ± SEM.
